# Supplementary material for: Content-rich biological network constructed by mining PubMed abstracts
Source: BMC Bioinformatics. 2004 Oct 8;5:147. doi: 10.1186/1471-2105-5-147 (PMC528731; doi:10.1186/1471-2105-5-147)
Supplement: Additional File 2 — The original results of the above study (non-essential files are deleted to keep the file size under the limit set by BMC bioinformatics). [file 1471-2105-5-147-S2.bz2 › chilibotAdditionalFile2/dip05/116ID8645696E460/html/ATPH_ATPC.html]

 


 **ATPH** and **ATPC** 
  
Found 201 abstracts in PubMed, retrieved 05.  
 

 What does Google say? 
 PDF only 
| .edu only 

---

**Interactive relationship** (e.g. stimulation, inhibition, etc)

**Neutral relationship**- Adipocyte lipid binding protein  [ **ATPC** ]   [ **ATPH** ]  ALBP or aP2 is an intracellular fatty acid binding protein which is found in adipocytes and macrophages and binds a large variety of intracellular lipids with high affinity.  Ref: 12794068 J Biol Chem< MedlineTA>J Biol Chem,

**Non-interactive relationship** (e.g. studied together, co-existance, homology, etc.)

- Apolipoprotein E apoE is a lipid binding protein  [ **ATPC** ]   [ **ATPH** ]  that plays an important role in tissue repair following brain injury.  Ref: 12793762 J Radiat Res Tokyo , 2002
- The zebrafish CRABP exhibits highest sequence identity to the pufferfish CRABPIIa 83% and CRABPIIb 79%, and human CRABPII 74% than to any other member of the intracellular lipid binding protein  [ **ATPC** ]   [ **ATPH** ]  ILBP family.  Ref: 12853146 Gene, 2003
- First, Notch1 activation by neuronal contact induces the glial expression of the brain lipid binding protein  [ **ATPC** ]   [ **ATPH** ]  BLBP and erbB2 genes.  Ref: 12853432 J Neurosci, 2003
- This is a 136 residue, 10 stranded, antiparallel beta barrel protein with seven beta hairpins and is a member of the intracellular lipid binding protein  [ **ATPC** ]   [ **ATPH** ]  iLBP family.  Ref: 12834350 Biochemistry, 2003
- Salt modulates the stability and lipid binding affinity of the adipocyte lipid binding protein  [ **ATPC** ]   [ **ATPH** ] .  Ref: 12794068 J Biol Chem< MedlineTA>J Biol Chem, 2003
